# Cases of Tedious Labour, in Which the Secale Cornutum Was Exhibited; with Remarks

**Published:** 1827-09

**Authors:** James Guthrie

**Affiliations:** Surgeon, Kilmarnock.


### MIDWIFERY.

### *Cases of Tedious Labour, in which the Secale Cornutum was exhibited; with Remarks.* By James Guthrie, Esq. Surgeon, Kilmarnock.

This remedy appears to have been used above half a century ago, though only occasionally since, to excite the uterus in cases of tedious labour arising from deficient pains. Its use being sometimes attended with dangerous effects, it soon fell into disrepute. It has, however, again been revived, and some examples have of late been published in favour of its reputed virtues.

The following cases, I think, very strikingly illustrate its efficacy.

Case I.—On Saturday morning, 20th May, 1826, I was requested to attend a married woman in her first labour. The liquor amnii had been discharged two hours previous to my arrival. The os uteri was found dilated to the size of half a crown; pains weak and inefficient, and recurring every fifteen minutes. I remained with her for an hour, expecting that, by dilating the os uteri gently during a pain, I might bring on more efficient action. In this, however, I was disappointed. I then left her, called occasionally during the day, but could not perceive any difference in the strength or frequency of the pains. I was sent for at eight o'clock in the evening; the pains appeared considerably stronger, but I was astonished to find the os uteri not much more dilated than in the morning. Pains frequent and apparently severe, but producing not the slightest effect in advancing the labour. In this state the labour continued until ten o'clock. The external as well as internal parts being in a favourable state for dilatation, I thought this might prove a fit case for a trial of the secale. I accordingly procured three scruple-doses, one of which I gave at fifteen minutes past ten. In twenty minutes after its exhibition, uterine action became sensibly stronger, and I was delighted to find the os uteri dilating and the head descending. The pains, which were formerly useless, were now strong and forcing, without almost any intermission. In fact, the uterus appeared to be aware (if I may so express myself,) that nothing would do but the complete expulsion of its contents. This took place in fifty minutes after the effects of the ergot became apparent, or in one hour and ten minutes after its exhibition. The placenta followed the expulsion of the child almost instantly. Mother and child did well.

Case II.—This woman's labour was ushered in by the sudden discharge of the liquor amnii; slight pains then commenced; and the waters continued to drain away for some time afterwards. In this state she lingered for thirty-six hours. A midwife was, however, employed in the interval, who remained with the woman a considerable time; but, on finding the labour tedious and the umbilical cord presenting, she desired a surgeon to be called. I was accordingly sent for: examination discovered nearly a foot of umbilical cord in the vagina, the pulsation of which had entirely stopped. The patient stated that the motion of the child had for some time ceased. The os uteri was somewhat dilated, but with difficulty reached; the head of the child, though very high up, was felt through the uterus, presenting; the pains were exceedingly feeble, and recurring at long intervals. I remained with the woman nearly three hours, during which time the pains were useless and inefficient, and not in the slightest degree advancing the labour. I therefore, without further procrastination, exhibited one scruple of the secale in cold water. In twenty minutes afterwards, the pains became more frequent, and longer in continuance. At this period a second scruple was given. The pains were now very severe, and almost constant, (this last effect is peculiar to the ergot;) the head of the child was evidently descending; the os uteri was nearly effaced. She complained of pain in the bowels, which was probably referable to the ergot; a delicate hint, no doubt, of its *modus operandi.* The pains continued so severe and uninterrupted, that I hesitated in giving more of the secale. The head of the child had descended into the hollow of the sacrum, and the umbilical cord before it. The patient had occasional attacks of vomiting.

After two or three hours, the pains began to fall off, and the expulsive efforts of the uterus were not so powerful. I gave at this period a third scruple, but it was instantly rejected, and I did not venture to give more. The labour now went on very slowly; pains recurring every fifteen minutes, and of short continuance; uterine action appeared to cease after a single expulsive effort; external parts very difficult to dilate. Delivery was at last accomplished, being a period of six hours after the exhibition of the ergot. The child was dead, and the cuticle in some places removed. Mother did well.

Before leaving this case, it may be asked, why was the ergot given in a case where the umbilical cord presented? I shortly answer, because I was convinced that the child was dead, from the flaccidity of, and want of pulsation in, the umbilical cord. The sensations of the mother likewise confirmed me in this opinion. Laying aside entirely the difficulty of turning a child in a uterus from which the waters had been so long evacuated, I consider that I was inflicting infinitely less suffering on the mother by the course pursued, than if I had attempted to turn the child. This case, though not so rapid as the former, indicated sufficiently the influence of the remedy over the action of the uterus. Indeed, had the ergot not been exhibited, the case, I am convinced, would have ended in instrumental labour.

Case III.—I was called to attend this woman about two o'clock P.M. on the 17th April, 1827. She had been in labour since the preceding evening. The pains were now very severe, but recurring at rather long intervals; the os uteri was considerably dilated, and the membranes protruding. In the course of two hours, the membranes gave way, and the head slowly descended into the hollow of the sacrum; but here it remained stationary, notwithstanding the frequency and severity of the pains. I prescribed, at seven P.M., one drachm of the ergot to be given in divided doses, as in former cases. Delivery took place in an hour thereafter. Mother and child did well.

Case IV.—I was requested, on the evening of the 8th of May,

1827, to attend Mrs. G— in her third labour. On my arrival, I found a midwife there, who had attended the patient for a period of three days. Examination discovered the body of the uterus presenting in the form of a bag low down in the vagina. The pains were extremely strong, and appeared more intent on the expulsion of the uterus than that of the child. Search was now made for the os uteri, but in vain. I therefore lost no time in introducing the hand into the vagina, which was pushed on in the direction of the sacrum: here I reached the os uteri near the brim of the pelvis, which I soon restored to its natural position. The woman expressed herself much relieved, and exclaimed that "all was right now!" The pains continued strong, and appeared to be efficient. I had considerable difficulty, however, during the pains, in preventing antiversion of the uterus from again taking place. This I was able to obviate by pressing the os uteri upwards and forwards with the finger during a pain. This method likewise rapidly assists the dilatation of the organ. I waited two hours and more, in expectation of delivery being soon accomplished. The os uteri was still very rigid, but so much dilated as nearly to permit the head to pass. The pains were evidently falling off, and the woman was becoming exhausted. Uterine action was now suspended by sixty drops of Tinct. Opii, after which she enjoyed refreshing sleep for three hours. At the end of this period, the pains again commenced, but were exceedingly feeble, and produced not the slightest effect in advancing the labour. From the exhausted state of the woman, I now lost all hopes of delivery being accomplished by the natural efforts. At this juncture (seven o'clock in the morning,) I ordered one drachm of Secale Cornutum to be infused in six ounces of boiling water, two ounces of which were given every twenty minutes. After the second dose, uterine action became sensibly increased; but it was not until the exhibition of the third dose that the wonderful virtues of the remedy became apparent. Uterine action now became excessive. The pains were strong and forcing, without any intermission. The expulsion of the child took place in an hour and a half after the use of the ergot was commenced. Mother and child did well.—Here again is a case where the forceps would in all probability have been required.

*Remarks*.—These cases, taken together, confirm, in the most satisfactory manner, the virtues of the ergot. Its effects were most striking: the action of the uterus was quite peculiar; it appeared as if urged on to discharge its contents, without a moment to recruit itself. This was particularly observed in Cases I. and IV. In these the expulsive efforts of the uterus were so rapid and powerful toward the end, that I was afraid of laceration of the perineum. In Case II. the effect of the ergot was not nearly so rapid as in the other cases. Perhaps, had I exhibited the third dose sooner, the result might have been different. Be this as it may, the peculiar power of the remedy over uterine action was beautifully displayed, and exhibited in every other respect similar phenomena. In all these cases, one drachm of the ergot appeared to be sufficient to ensure its specific effects. Its virtues demand a place for it amongst the nervous excitants. This peculiar power appears to be primarily exerted on the stomach, and from thence communicated to the uterus through that mysterious channel, nervous agency.

Should the ergot be found, by further experience, to be so efficacious in cases of tedious labour, a great improvement will be made in the practice of midwifery. For, armed with it on the one hand, and the lancet on the other, no labour will ever be tedious, except those requiring manual assistance. The use of the forceps must now give place to a remedy so efficient, and so perfectly safe both to mother and child. But this, like every other excellent remedy, is apt to suffer abuse, and may, as formerly, do harm, if indiscriminately employed. The death of the child, and hemorrhage after delivery, were the evil consequences formerly ascribed to its exhibition. I do not deny but that the first of these might happen as already hinted; but that the second should occur, is altogether beyond my comprehension. The very reverse should be the case: for, if the remedy be capable of exciting the uterus to more powerful contraction during labour, it must certainly prove the very best security against the occurrence of hemorrhage after delivery.

*Kilmarnock; May 21st,* 1827.

